# Regulation of rDNA Transcription by Proto-Oncogene PELP1

**DOI:** 10.1371/journal.pone.0021095

**Published:** 2011-06-13

**Authors:** Vijay K. Gonugunta, Binoj C. Nair, Rajib Rajhans, Gangadhara R. Sareddy, Sujit S. Nair, Ratna K. Vadlamudi

**Affiliations:** 1 Department of Obstetrics and Gynecology, and Cancer Therapy and Research Center, University of Texas Health Science Center, San Antonio, Texas, United States of America; 2 Department of Molecular Medicine, University of Texas Health Science Center, San Antonio, Texas, United States of America; University of Pennsylvania, United States of America

## Abstract

**Background:**

Proline-, glutamic acid-, and leucine-rich protein (PELP1) is a novel nuclear receptor coregulator with a multitude of functions. PELP1 serves as a scaffolding protein that couples various signaling complexes with nuclear receptors and participates as a transcriptional coregulator. Recent data suggest that PELP1 expression is deregulated in hormonal cancers, and that PELP1 functions as a proto-oncogene; however, the mechanism by which PELP1 promotes oncogenesis remains elusive.

**Methodology/Principal Findings:**

Using pharmacological inhibitors, confocal microscopy and biochemical assays, we demonstrated that PELP1 is localized in the nucleolus and that PELP1 is associated with the active ribosomal RNA transcription. Cell synchronization studies showed that PELP1 nucleolar localization varies and the greatest amount of nucleolar localization was observed during S and G2 phases. Using pharmacological compounds and CDK site mutants of PELP1, we found that CDK's activity plays an important role on PELP1 nucleolar localization. Depletion of PELP1 by siRNA decreased the expression of pre-rRNA. Reporter gene assays using ribosomal DNA (pHrD) luc-reporter revealed that PELP1WT but not PELP1MT enhanced the expression of reporter. Deletion of nucleolar domains abolished PELP1-mediated activation of the pHrD reporter. ChIP analysis revealed that PELP1 is recruited to the promoter regions of rDNA and is needed for optimal transcription of ribosomal RNA.

**Conclusions/Significance:**

Collectively, our results suggest that proto-oncogene PELP1 plays a vital role in rDNA transcription. PELP1 modulation of rRNA transcription, a key step in ribosomal biogenesis may have implications in PELP1-mediated oncogenic functions.

## Introduction

Ribosomes are the protein synthesizers of the cell. Cell growth and proliferation is critically dependent on proper synthesis and functioning of ribosomes. Deregulation of ribosomal biogenesis has been linked to several diseases including cancer [1]. Emerging data implicate the activity of certain oncogenes [2] and tumor suppressor proteins [3] correlate with precise regulation of ribosome processing. For example, the oncogene cMyc, is shown to initiate and maintain tumorigenesis partly due to its ability to regulate ribosomal genes [4], [5]. Enhanced production of the ribosome and alteration in the ribosome structure are associated with neoplastic transformation [6]. However, molecular mechanism(s) by which oncogenes/tumor suppressors regulates ribosomal biogenesis remain elusive and is an active area of investigation.

The nucleolus is a distinct subnuclear compartment, where tandem repeated ribosomal RNAs (rRNAs) 18S, 5.8S and 28S are transcribed by RNA polymerase I (Pol I) as a single precursor, which is processed and assembled with the 5S rRNA (transcribed by RNA Pol III) and ribosomal proteins into ribosome subunits [7]. Ribosome biogenesis and ribosomal DNA (rDNA) transcription are closely correlated and it is suspected that rDNA transcription is under tight epigenetic control [8]. Recent proteomic analysis of purified nucleoli have identified ∼700 proteins with diverse functions, suggesting the complexity of nucleolar machinery [9]. Furthermore, interactions between factors involved in ribosome synthesis and specific steps in the cell division cycle are reported [10]. Transcription of rDNA is modulated during cell cycle progression, being low in early G1-phase, reaching highest levels in S- and G2-phases, and being shut off in mitosis [11]. Inhibition of cell cycle dependent kinases (CDKs) in interphasic cells hampers proper pre-rRNA processing and induces a dramatic disorganization of the nucleolus, implicating a regulatory role of CDK activities in ribosome biogenesis [12]. Molecular mechanisms by which cell cycle machinery cross-talk and regulate ribosomal biogenesis remain poorly understood.

Recently, we identified proline-, glutamic acid-, leucine-rich protein-1 (PELP1), as a novel substrate of CDKs [13]. PELP1 interacts with the cell cycle switch protein retinoblastoma (pRb) [14], PELP1 phosphorylation is regulated during cell cycle progression [13] and PELP1 over-expression promotes G1-S progression [14]. PELP1 is also recently identified as a proto-oncogene [15] that exhibits aberrant expression in many hormone-related cancers [16] and is a prognostic indicator of shorter breast cancer-specific survival and disease-free intervals when over-expressed [17]. PELP1 interacts with a number of nuclear receptors, functions as a scaffolding protein, and modulates the activities of several chromatin-modifying enzymes [18], but the mechanism(s) by which PELP1 promotes oncogenesis remains elusive.

In the present study, we demonstrated that PELP1 localizes to the nucleolar compartment in a cell cycle-dependent manner and that CDK phosphorylation modulates PELP1 localization to the nucleolar compartment. Our results suggest that PELP1 facilitates cell cycle machinery cross-talk with nucleolar machinery and plays an essential role in rDNA transcription.

## Results

### PELP1 exhibits nucleolar localization

**Figure 1:**
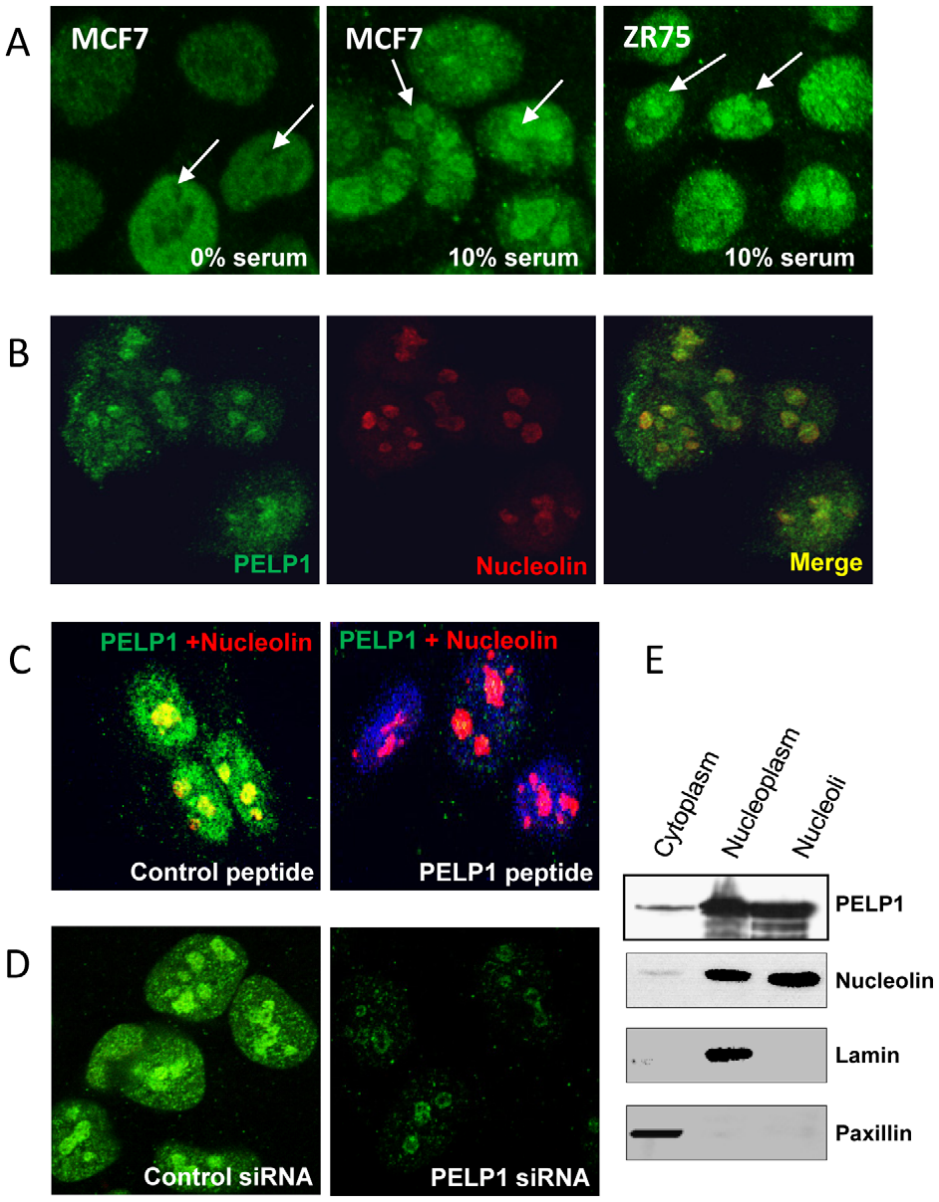
PELP1 localizes to the nucleolus. (**A**) MCF7 and ZR-75 cells were serum starved for 2 days and stimulated with 10% serum for 12 h. PELP1 (green) localization was visualized by using confocal microscopy. (B) HeLa cells were grown in 10% serum and the localization of PELP1 (green), the nucleoli marker nucleolin (red) and colocalization (yellow) was analyzed by using confocal microscopy. (**C**) PELP1 localization was analyzed by using a control peptide or PELP1 epitope-specific peptide adsorbed antibody. (**D**) HeLa cells were transfected with either control or PELP1-specific siRNA and the localization of PELP1 (green) was analyzed by using confocal microscopy. (**E**) HeLa cells were fractionated into cytoplasm, nucleoplasm and nucleoli, and the presence of PELP1 in these fractions was analyzed by immunoblotting. Paxillin, lamin and nucleolin were used as cytoplamic, nuclear and nucleoli markers respectively.

During our ongoing investigations examining the role of PELP1 in cell cycle progression using immunofluorescence staining analysis, we discovered that PELP1 uniquely localized in the nucleolus. MCF7, a breast cancer cell line, when synchronized to G1 phase by serum starvation, PELP1 had prominent nuclear localization with negligible nucleolar localization (Figure 1A, left panel). However, when these cells were allowed to progress through cell cycle with addition of 10% serum, PELP1 exhibited prominent nucleolar localization (Figure 1A, middle panel). Similar results were observed in another breast cancer cell line ZR-75 (Figure 1A, right panel). To examine this further, we used HeLa cells, model cells commonly used for cell cycle studies. Confocal studies using nucleolin, a well-established, positive nucleolar marker confirmed PELP1 localizes to nucleoli (Figure 1B). Competition assays using a peptide that comtains epitope to the PELP1 antibody (Figure 1C) and down regulation of PELP1 using siRNA (Figure 1D) substantially reduced PELP1 nucleolar localization further confirming the authenticity of PELP1 nucleolar localization. Biochemical fractionation results also supported the localization of PELP1 to the nucleolar compartment (Figure 1E). Collectively, these results suggest that a part of total cellular PELP1 exhibits nucleolar localization.

### PELP1 nucleolar localization associates with active rDNA transcription

**Figure 2:**
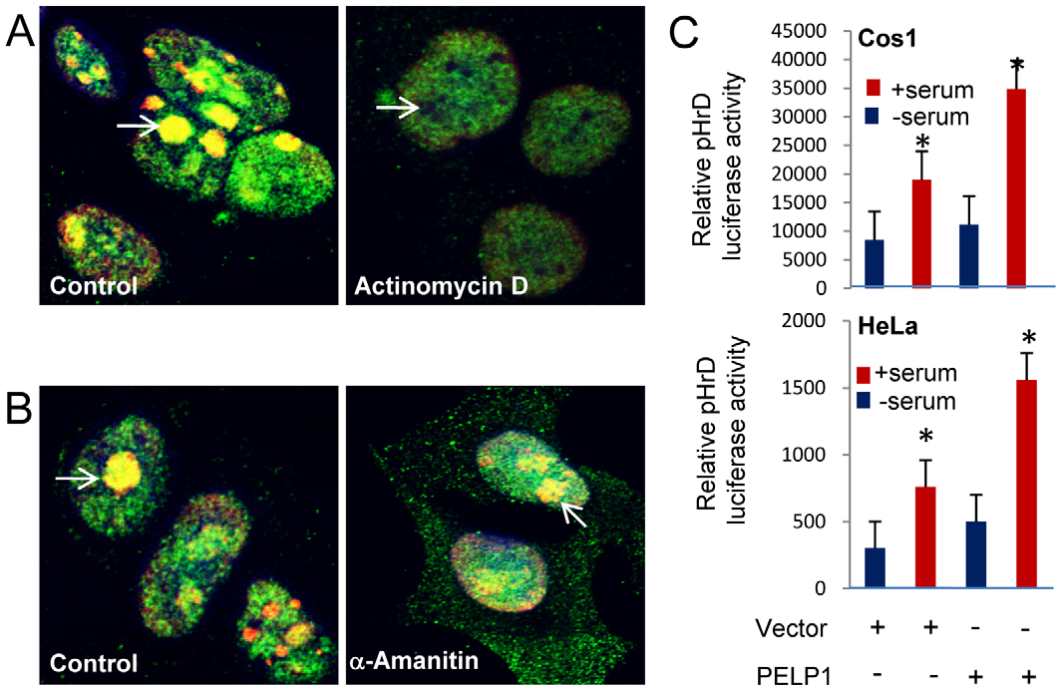
PELP1 nucleolar localization depends on active rDNA transcription. HeLa cells were treated with or without actinomycin D (Pol I transcription inhibitor, 4 µg/ml for 12 h) [**A**] and with or without α-amanitin (Pol II transcription inhibitor 80 µg/ml for 12 h) [**B**]. The localization of green (PELP1), red (nucleolin) and yellow (colocalization of PELP1 with nucleolin) was analyzed by using confocal microscopy. (**C**) HeLa or Cos1 cells were transfected with pHrD Luciferase reporter vector along with control or PELP1-expressing vectors. After 6 h, cells were serum starved for 24 h and then stimulated with 10% serum for 24 h and the reporter gene activity was measured. Results are the average of 3 independent experiments. *p-value <0.05.

We next examined whether PELP1 nuclear localization correlates with Pol I-mediated ribosomal transcription. Treatment of cells with actinomycin D at concentrations that inhibit the rDNA transcription by Pol I, abolished PELP1 localization to the nucleolus (Figure 2A), while control proliferating cells had PELP1 accumulation in the nucleolus. On the other hand, treatment of cells with α-amanitin, a Pol II transcription inhibitor, did not have any detectable effect on the localization of PELP1 to the nucleolus (Figure 2B). These results suggested that PELP1 localization coincided with ribosomal transcription activity by Pol I. Since PELP1 functions as coregulator of several nuclear receptors such as ER and E2F, we examined whether PELP1 enhances the activity of human rDNA-promoter luciferase reporter (pHrD-IRES-Luc). Co-transfection of PELP1, but not control vector, significantly enhanced the serum-mediated increase in the rDNA promoter activity both in Cos1 and HeLa cells (Figure 2C). Dependence of PELP1 localization on functional Pol I activity and the ability of PELP1 to enhance the rDNA-promoter luciferase reporter suggests that PELP1 may be involved in the rDNA transcription.

### PELP1 nucleolar domains are important for PELP1 nuclear localization and participation in rDNA transcription

**Figure 3:**
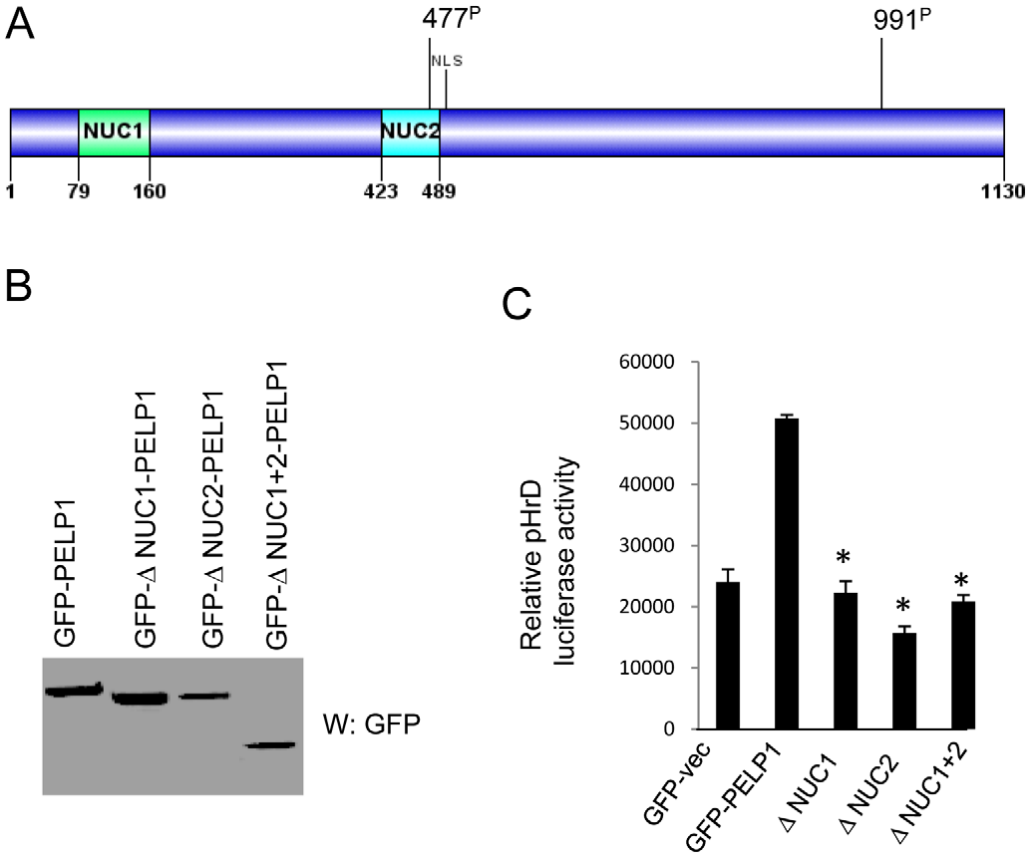
PELP1 activation of ribosomal promoter depends on functional nucleolar domains. (**A**) Schematic representation of PELP1 nucleolar domains. (**B**) 293T cells were transiently transfected with PELP1 WT and Δ-Nuc-PELP1 mutant vectors. Expression of the constructs was analyzed by immunoblotting. (**C**) 293T cells were transfected with pHrD luciferase reporter along with PELP1 WT or Δ-Nuc-PELP1 vectors. Cells were serum starved for 24 h and then stimulated with 10% serum for 24 h and the reporter gene activity was measured. Results are the average of 3 independent experiments. Statistical analysis, paired *t*-test was performed and considered significant when *p-value *<0.05.

Homology search using a bioinformatic approach revealed that PELP1 contains two nucleolar domains [19] that are commonly present in many proteins that localize in the nucleolus. These domains are localized in the N-terminal region of PELP1 comprising amino acids 79–160 (Nuc 1) and 423–489 (Nuc 2) (Figure 3A). To examine the significance of these domains in PELP1-mediated coactivation of ribosomal promoter, we deleted these two regions from full-length PELP1. Western analysis revealed expression of mutants and their migration to the expected sizes (Figure 3B). In reporter gene assays, PELP1 lacking nucleolar domains failed to activate the ribosomal promoter reporter, while PELP1WT enhanced the ribosomal promoter activity (Figure 3C). These results suggested that functional nucleolar domains in PELP1 are important for nuclear localization and ribosomal promoter activation.

### PELP1 localization in the nucleolus is cell cycle dependent

**Figure 4:**
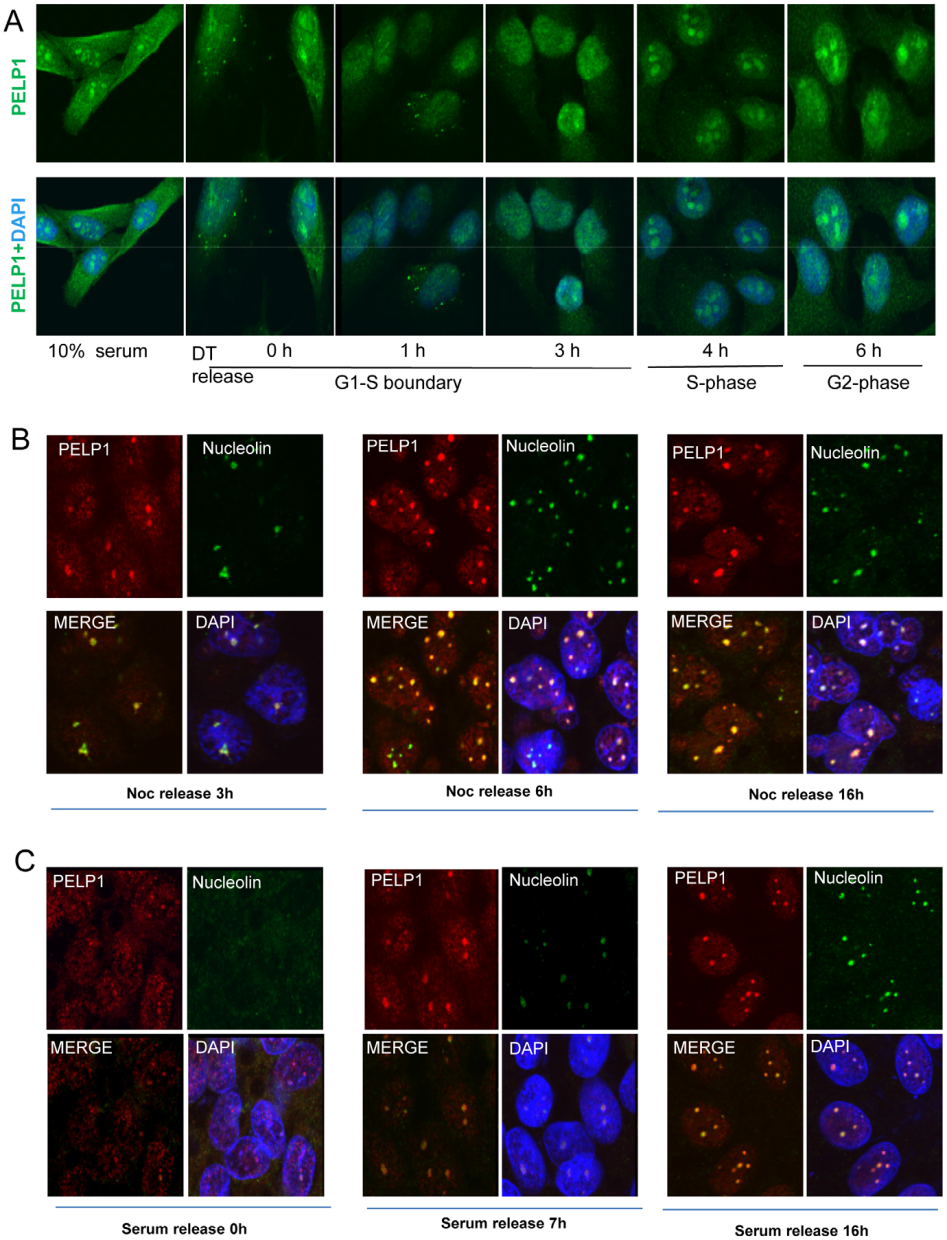
PELP1 nucleolar localization depends on cell cycle progression. (**A**) HeLa cells were arrested in G1-S boundary by a double thymidine block and cells were released to progress into the S and G2 phases of the cell cycle. PELP1 localization was visualized by using confocal microscopy. (**B**) ZR-75 cells were synchronized to G2/M phase using nocodazole (15 µM) for 18 h and released to progress into the cell cycle by addition of 10% serum. PELP1 localization was monitored with confocal microscopy by colocalizing with nucleolin. (**C**) ZR-75 cells were synchronized to G1 phase by serum starvation and released to progress into the cell cycle by addition of 10% serum. PELP1 localization was monitored by using confocal microscopy in different time intervals by colocalizing with nucleolin.

The intracellular localization of PELP1 appears to be a dynamic process with only 50–60% of the asynchronized cell population having more preponderance of PELP1 in the nucleolus. Intriguingly, PELP1 localization in the nucleolus appears to correlate with the number and size of the nucleolus in the cells. This raised an interesting possibility that PELP1 localization in the nucleolus is regulated by cell cycle phases. To address this hypothesis, we synchronized HeLa cells in G1-S boundary using a well-established double-thymidine block protocol and then released the cells into the cell cycle [20]. Cell cycle phases at each time points were analyzed by flow cytometry (data not shown) and depicted below each lane (Figure 4A). In the G1-S boundary, a very limited amount of PELP1 was localized in the nucleolus but upon release into the cell cycle phases by addition of thymidine-free medium for 1 or 3 h, PELP1 localization in the nucleolus gradually enhanced. After 4 h when the cells moved into S-phase, both the number of nucleoli and PELP1 staining in the nucleolus greatly enhanced. After 6 h (when the cells were in the G2 phase), many smaller nucleoli coalesced to form a larger nucleolus structure and PELP1 staining was at a maximum, coinciding with nucleolar reorganization. To confirm this cell cycle-dependent intracellular localization of PELP1, we performed additional cell synchronization to mitosis by using nocadazole treatment and a mitotic shake off protocol [21]. Cells were re-plated on sterile coverslips and released into the cell cycle by using nocadazole-free medium. Confocal analysis using a nucleolar marker had expected results including the lack of a nucleosome in mitosis, multiple nucleoli in G1/S phase and big nucleoli in G2 phase. Interestingly, PELP1 also exhibited localization similar to the nucleoli marker nucleolin (Figure 4B). We also verified cell cycle dependence of PELP1 localization to the nucleoli by using serum starvation-mediated synchronization and release assays (Figure 4C). Together, these results suggest that PELP1 nucleolar localization occurred depending on cell cycle progression.

### CDK phosphorylation regulates PELP1 nucleolar localization

**Figure 5:**
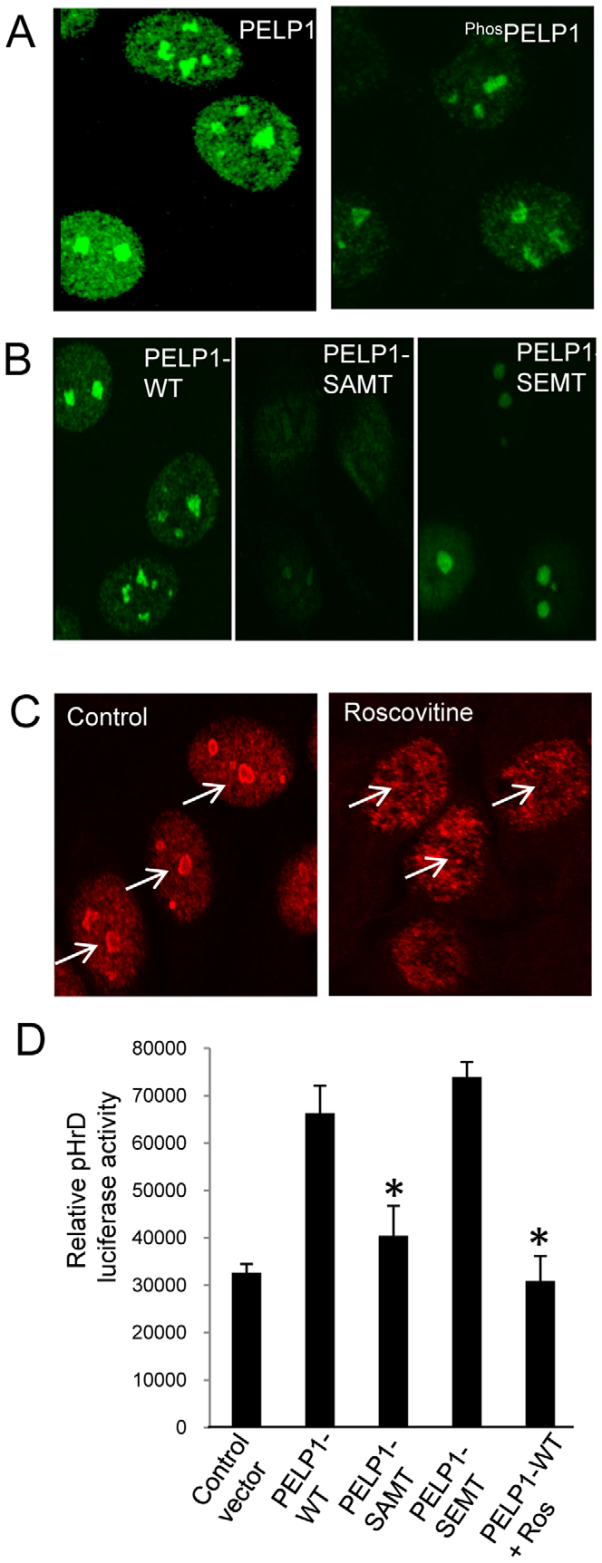
CDKs regulate PELP1 nucleolar localization. (**A**) ZR-75 cells at sub confluence were fixed and the localization of ^phos991^PELP1was analyzed by using confocal microscopy. (**B**) Localization of GST-tagged PELP1 or PELP1 S991AMT or PELP1 S991EMT were visualized by using confocal microscopy with the antibody against the GST epitope in ZR-75 cells. (**C**) PELP1 was visualized in ZR-75 cells after serum starved for two days and released with 10% serum in absence or presence of roscovitine (10 µM) by using confocal microscopy. (**D**) 293T cells were transfected with pHrD luciferase reporter vector along with PELP1 WT or PELP1 S991AMT or PELP1 S991EMT expressing vectors. Cells were serum starved for 24 h and then stimulated with 10% serum for 24 h and the reporter gene activity was measured. Results are the average of 3 independent experiments. *p-value <0.05.

PELP1 was recently identified as a novel substrate of CDKs [13]. We therefore examined whether CDK signaling regulates PELP1 localization to the nucleolus by using a phospho-PELP1 Ser991 antibody that uniquely recognizes CDK-phosphorylated PELP1 [13]. Confocal analysis revealed that substantial amount of phospho-PELP1 localized to the nucleolus (Figure 5A). Mutation of the CDK sites (477 and 991) in PELP1 from Ser to Ala (PELP1SAMT) affected PELP1 localization in the nucleoli, while mutation of Ser to Glu (PELP1SEMT that mimics phosphorylation) did not affect PELP1 localization to nucleolus (Figure 5B). Pharmacological inhibition of CDKs using roscovitine also inhibited PELP1 localization to the nucleolus (Figure 5C). In reporter genes assays, PELPWT and the PELP Ser-to-Glu mutant activated pHrD promoter reporter (Figure 5D). PELP1 Ser-to-Ala mutation or roscovitine treatment abolished the PELP1-mediated increase in pHRD reporter gene activation. Collectively, these results suggest that CDK2 regulated PELP1 localization to the nucleolus and CDK phosphorylation was needed for optimal activation of the ribosomal promoter by PELP1.

### PELP1 recruits to rDNA promoter and is needed for optimal transcription of rDNA

**Figure 6:**
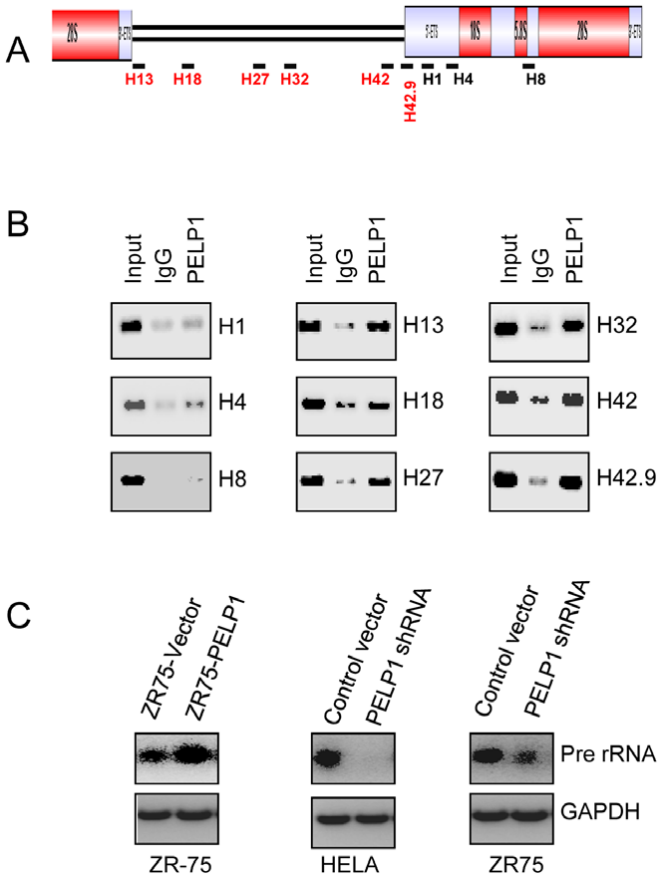
PELP1 associates with the rDNA promoter and regulates rDNA transcription. (**A**) Schematic representation of the rDNA gene and primer pairs used in the ChIP assay. (**B**) ChIP assay was done using antibodies specific for PELP1 or isotype rabbit IgG control in ZR-75 cells. DNA recovered from ChIP or input controls was subjected to conventional PCR using the indicated primers spanning the promoter and coding regions. (**C**) Total RNA was isolated from ZR-75 overexpressing PELP1 and HeLa, ZR-75 cells expressing control or PELP1 siRNA analyzed for the status of pre-rRNA transcription by RT-PCR.

Earlier studies showed that PELP1 facilitate chromatin modifications by recruiting to target gene promoters [22]. We therefore examined whether PELP1 was directly involved in the rDNA transcription by recruiting to the rDNA promoter (Figure 6A). Previous studies identified several regulatory regions in the rDNA promoter [23], which are schematically represented in Figure 6A. Chromatin immunoprecipitation studies revealed that PELP1 was efficiently recruited to the promoter region of the rDNA (Figure 6B). Further analysis using additional primers showed efficient recruitment of PELP1 at various known regulatory sites in the rDNA promoter (Figure 6B) with no or weak recruitment to the coding regions. Accordingly, overexpression of PELP1 in ZR-75 cells had increased rRNA transcription as measured by RT-PCR analysis. On the other hand, PELP1 knock-down in ZR-75 and HeLa cells resulted in substantially reduced rRNA transcript levels (Figure 6C). Collectively, these results suggest that PELP1 has potential to recruit to the rDNA promoter and can modulate pre-rRNA transcript levels.

## Discussion

The nucleolus is a sub-nuclear organelle that plays a vital role in ribosome biogenesis. To drive the cell cycle progression, an adequate ribosomal number is necessary to meet the needs of ongoing protein synthesis. Deregulation of ribosomal biogenesis leads to tumorigenesis [1]. PELP1 is a proto-oncogene that is implicated in hormonal tumor progression [24]. In this study, we found that (i) PELP1 localized to the nucleolar compartment, (ii) PELP1 localization changed during cell cycle progression, (iii) CDKs phosphorylation modulated PELP1 nucleolar localization, (iv) PELP1 recruited to the ribosomal promoter, and modulated ribosomal reporter activity, and (v) PELP1 was needed for optimal rRNA transcription. Collectively, these results suggest that PELP1 plays a vital role in rDNA transcription, and thus may contribute to tumorigenesis by accelerating ribosomal biogenesis.

Three multi-subunit RNA polymerase-coordinated function is involved in ribosome synthesis. Pol I synthesizes the 18S, 5.8S and 28S, Pol II synthesizes mRNA of genes involved in ribosomal biogenesis and Pol III facilitates production of 5S rRNA [2]. Pol I is restricted to the nucleolus and is involved in pre-rRNA synthesis. A number of transcription factors and coregulators, such as UBF and SL1, are shown to localize to the nucleolus and regulate Pol I transcription [25]. Our results demonstrate that PELP1 localizes to the nucleolus. Inhibition of Pol I transcription but not Pol II transcription abolished PELP1 nucleolar localization. Further, siRNA-mediated knock-down of PELP1 substantially affected the synthesis of pre-rRNA. PELP1 functions as a coregulator of many nuclear receptors, such as ER and AR, and transcription factors including E2F1 and STAT [18]. PELP1 recruitment to the nucleolus during stages of active Pol I transcription, and its ability to regulate ribosomal promoter activity and rRNA expression suggest that PELP1 has potential to function as a coregulator of Pol I transcription.

CDKs are the master regulators of cell cycle progression and their expression is deregulated in tumors, indicating that phosphorylation of downstream effector proteins by CDKs is vital in tumorigenesis [26]. PELP1 was recently identified as a novel substrate for the CDKs [13]. Our results suggest that functional CDK activity is necessary for PELP1 localization to the nucleolus as pharmacological inhibition of CDKs using roscovitine abolished PELP1 localization to the nucleolus. Further, mutation of the CDK phosphorylation site in PELP1 (Ser 477, 991 to Ala, impaired the localization of PELP1 to the nucleolus. Complementing these results, both roscovitine treatment and/or mutation of CDK1 sites in PELP1 decreased the PELP1-mediated increase in the activation of the ribosomal promoter reporter. Collectively, these results suggest that optimal CDK activity is necessary for PELP1 nucleolar localization and modulation of rDNA transcription.

PELP1 can interact with and modulate function of several chromatin-modifying enzymes including histone-modifying acetylases and deacetylases [27] and histone-modifying methyl-transferases and demethylases. PELP1 is a component of the MLL1 methyltransferase complex [28], and PELP1 functions as a reader of dimethyl-modified histones [22]. Our ChIP results demonstrated that PELP1 was recruited to the different promoter regions of the rDNA and enhanced the transcription of pre-rRNA. The ability of PELP1 to promote chromatin modifications and the ability of CDK to modulate PELP1 localization to nucleolus, suggest that PELP1 may serve as a key coregulator that connects CDK signaling to the regulation of Pol I-mediated transcription of rDNA genes probably by facilitating epigenetic changes, which will be addressed in future studies. A recent paper reported that PELP1 associates with the SENP3-associated complex comprising PELP1, TEX10 and WDR18, and is involved in maturation and nucleolar release of the large ribosomal subunit [29]. Collectively, these emerging findings strongly support a nucleolar function for PELP1 and that PELP1 may be involved in multiple steps of ribosomal biogenesis.

Recent studies that profiled nucleolar proteins using global quantitative proteomics approach identified PELP1 as one of the components of the nucleolus [9], [30] and these findings further support our observation that PELP1 localized to the nucleolus. Most of the nucleolar proteins appear to contain nucleolar domains, which may have important roles in the participation of the rDNA transcription, processing of the pre-rRNA or other functions of the nucleolus [19]. Evolving evidence suggests that PELP1 function as a large scaffolding protein, modulating gene transcription with protein-protein interactions [16]. Protein domain homology analysis using NIH BLAST/Uniprot.org program revealed that PELP1 contains two nucleolar domains (Nuc 1 and Nuc 2). Deletion of these domains from PELP1 impaired activation of rDNA transcription, suggesting that PELP1 nucleolar domains have important roles in rDNA transcription.

Many proto-oncogenes [31] and tumor suppressors cross-talk during ribosomal biogenesis [3], [32] and some proto-oncogenes directly regulate ribosomal biogenesis by binding to the promoter regions of the rDNA and modulating RNA polymerase 1-mediated transcription [4], [5]. PELP1 is proto-oncogene that exhibits deregulated expression in hormonal cancers [15], and is shown to promote tumorigenesis by accelerating cell cycle progression [13]; however the molecular mechanism by which PELP1 achieves these functions had been elusive. We now show that PELP1 localizes to the nucleolar compartment in a cell cycle stage-dependent manner and its dependence on CDK activity suggests that PELP1 participates as a sensor of CDK-mediated signaling and facilitates optimal ribosomal RNA synthesis.

In summary, our results demonstrate that proto-oncogene PELP1 localization to the nucleolus is mandatory for optimal rDNA transcription and functional CDK2 activity plays a positive role in PELP1 translocation to nucleolus and in participation of rDNA transcription. On the basis of these findings, we speculate that PELP1 modulates the rDNA transcription and thus contributes towards tumorigenesis by accelerating cell cycle progression.

## Materials and Methods

### Cell lines and reagents

Human breast cancer cells MCF7, ZR-75, Cos1, HeLa and 293T cells were obtained from American-Type Culture Collection (ATCC, Manassas, VA). PELP1 antibody was purchased from Bethyl lab (Montgomery, TX). Anti-GFP antibody was purchased from Clontech (Mountain View, CA). PELP1 SMARTpool siRNA were purchased from Dharmacon (Lafayette, CO). Nucleolin and GST antibodies were purchased from Sigma (St. Louis, MO) and Upstate (Chicago, IL) respectively. Phospho-PELP1 antibody was generated by our lab against phospho-PELP1 Serine 991 (peptide sequence TLPPALPPPE(pS)PPKVQPEPEP) [33].

### Generation of PELP1 mutants lacking nucleolar domains

The PELP1 mutant that lacks the nucleolar domains 1 and 2 were generated on either pEBG-PELP1 or GFP-PELP1 [34] using the overlapping-PCR protocol. The primer sequences used include: F1, TCTCGAGCTCAAGCTGCGGC AGCCGTTCTGAGTG; R1, CAGCTGGGCTGCATACGACCCATGCAGCCGCAA; F2, CTGCATGGGTCGTATGCAGCCCAGCTGCCTG; R2, GGCGCTAGGCTTC TCTCCTGGCCTGGAGA; F3, GGCCAGGAGAGGAAGCCTAGCGCCCCCAAG; R3, CCGCGGTACCGTCGACTAGGAGTCAGGCTCTGTG.

### Reporter gene assays

Human rDNA-Luciferase Vector (pHrD-IRES-Luc) plasmid that contains the rRNA promoter spanning −410 to +314 bp was from Dr. Samson Jacob [35]. Reporter gene assays were performed by transient transfection using FuGENE6 (Roche Indianapolis, IN ) as described [36]. Briefly, cells were transfected using 1 µg of pHrD-IRES-Luc reporter, 10 ng psv β-gal, with or without 100 ng of PELP1 wild-type (WT) or various PELP1 mutant (MT) expression vectors. Cells were lysed in passive lysis buffer 36–48 h after transfection, and the luciferase assay was performed using a luciferase assay kit (Promega, Madison WI). Each transfection was carried out in 6-well plates in triplicate and normalized with either β-gal activity or the total protein concentration.

### Cell extracts, Western blots, and immunoprecipitation

Cell lysates for Western blot analysis were prepared as described [33]. Nucleolar isolation and protein extracts were prepared using the protocol as described [37].

### Chromatin immunoprecipitation

The chromatin immunoprecipitation (ChIP) analysis was performed as described previously [38]. Briefly, ZR-75 cells were cross-linked using formaldehyde, and the chromatin was subjected to immunoprecipitation with the respective antibodies. Isotype-specific IgG was used as a control. DNA was re-suspended in 40 µl of TE buffer and used for PCR amplification with the specific primers described in earlier published study [23].

### Cell Synchronization, Immunofluorescence and Microscopy

Cells were synchronized to G0/G1 phase by serum deprivation for 3 days and released into the cell cycle by addition of 10% FBS-containing medium as described [33]. Cell cycle synchronization in G1-S boundary was performed by using a double thymidine block protocol. Cells were plated at low confluence on sterile glass slides and treated with 2 mM thymidine-containing medium for 16 h. The medium was then removed and the cells were washed with PBS and treated with thymidine-free medium for another 9 h. The cells were further treated with medium containing 2 mM thymidine for additional 16 h to arrest majority of cells in G1-S boundary. Cells were finally washed with PBS and released into the cell cycle using normal cell growth medium for various periods of time intervals. Cells were fixed in 4% paraformaldehyde, permeabilized with 0.2% Triton X-100 in PBS and subjected to immuno-staining with PELP1 (Bethyl lab), PELP1 991P [33] and nucleolin (Upstate or Sigma-Aldrich) antibodies (1∶500). Images were acquired using confocal microscopy (Olympus FV-1000) [39]. Cellular localization of PELP1 or PELP1 991P and nucleolin was determined by confocal microscopy as described previously [38].
